# Data on nephroprotective effect of all-trans retinoic acid in early diabetic nephropathy

**DOI:** 10.1016/j.dib.2018.08.080

**Published:** 2018-08-29

**Authors:** Edith Sierra-Mondragón, Eduardo Molina-Jijón, Carmen Namorado-Tónix, Rafael Rodríguez-Muñoz, José Pedraza-Chaverri, José L. Reyes

**Affiliations:** a Departamento de Fisiología, Biofísica, y Neurociencias, Centro de Investigación y Estudios Avanzados del Instituto Politécnico Nacional (CINVESTAV-IPN), México CDMX 07360, Mexico; b Glomerular Disease Therapeutic Laboratory, Department of Internal Medicine, Rush University Medical Center, Chicago, Illinois, United States; c Facultad de Química, Departamento de Biología, Universidad Nacional Autónoma de México (UNAM), México CDMX 04510, Mexico

**Keywords:** Diabetic nephropathy, Inflammation, All-trans retinoic acid, Cytokines

## Abstract

Data showed in this report are related to the research article entitled “All-trans retinoic acid ameliorates inflammatory response mediated by TLR4/NF-кB during the initiation of diabetic nephropathy” by Sierra-Mondragon et al. (2018) [1]. Diabetic nephropathy (DN) has become the main cause of renal failure. Inflammatory molecules such as cytokines, chemokines and growth factors play a key role in DN-induced renal injury Pichler et al. (2016) [2]. Results illustrate the effect of all-trans retinoic acid (ATRA), an active metabolite of vitamin A, on the renal alterations related to diabetes, among them glomerular and tubular dysfunction, and its effect on renal inflammation in different nephron segments: glomeruli, proximal and distal tubules in an initial stage of DN. Data were obtained by physical-biochemical measurements and Western blot assays performed on isolated glomeruli, proximal and distal tubules from rat kidneys.

**Specifications Table**

**Table:** 

Subject area	*Biology*
More specific subject area	*Immunology and inflammation*
Type of data	*Figures and Tables*
How data was acquired	*Renal function parameters were detected by a spectrophotometric method (Spectrophotometer Infinite M200, Tecan; Männedorf Suiza) and chemiluminescent Western blot were detected in an EC3 Imaging System (UVP BioImaging Systems, Cambridge, UK). Protein band densities were quantified by transmittance densitometry.*
Data format	*Processed data and raw data*
Experimental factors	*To analyze protective effects of ATRA, 4 experimental groups were evaluated; Control (CTL), Diabetic (DBT), Diabetic rats treated with ATRA (DBT+ATRA) and control rats treated with ATRA (ATRA).*
Experimental features	*Western blot analyses were performed on isolated glomeruli, proximal and distal tubules of rat kidneys from the four experimental groups.*
Data source location	*Mexico City, México*
Data accessibility	*Data are available in this paper*

**Value of the data**

• The data show the nephroprotective effect of ATRA, leading to preservation of renal function by suppressing inflammation, in early stages of streptozotocin-induced diabetes in rats.
• These data are useful as there are few reports on the anti-inflammatory protective effects of ATRA on early DN. The observed beneficial effects might represent a therapeutic alternative to reduce the progression of DN, which is one the pathologies leading to end stage renal disease worldwide.
• In addition, these data may be relevant for (i) other researchers using ATRA in their studies since at low doses we used (1 mg/kg), it did not show untoward effects and (ii) we provide experimental protocols for isolation of different nephron segments: glomeruli, proximal and distal tubules by Percoll gradients and sieving, without microdissection. The data show the nephroprotective effect of ATRA leading to preservation of renal function by suppressing inflammation in early stages of streptozotocin-induced diabetes in rats.

## 1 Data

**Fig. 1:**
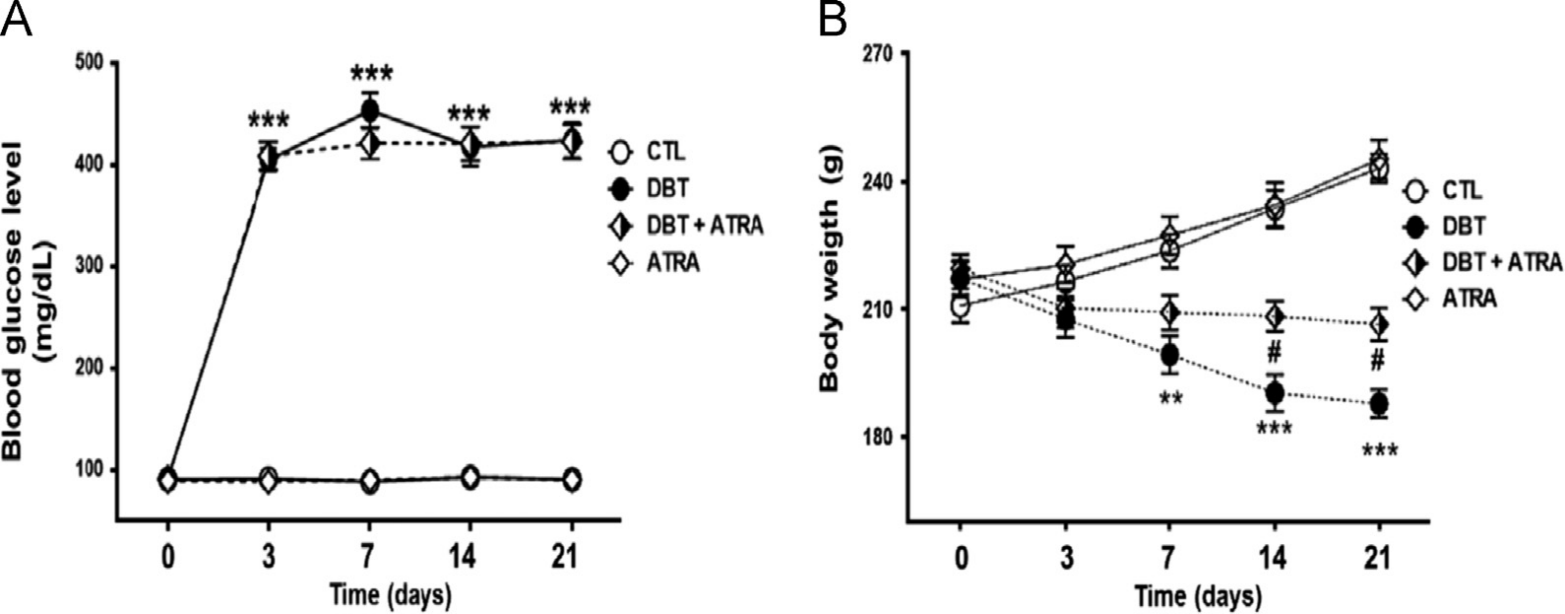
Effect of ATRA in early diabetic characteristics such as (A) Blood glucose levels, (B) Body weight. Data are mean±SD from 12 rats per group. Significance of difference; *p< 0.05, **p< 0.001 ***< 0.001 vs CTL group; # *P*< 0.05, vs DBT group.

**Fig. 2:**
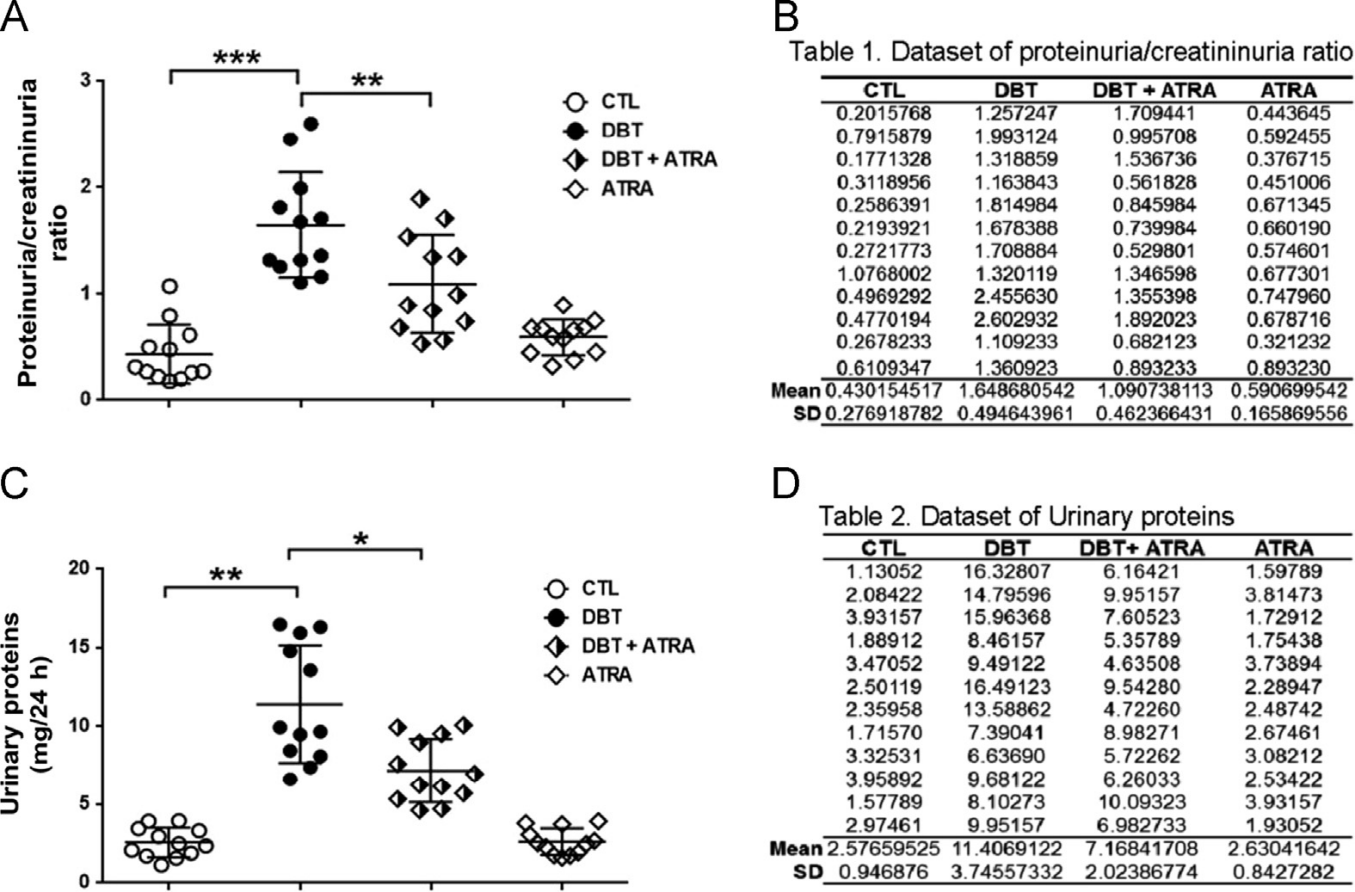
The effect of ATRA in glomerular damage during initial DN was assessed by (A) Proteinuria/creatininuria ratio and (C) Urinary proteins, (B and D) Raw data used for graphical. Data are mean±SD from 12 rats per group. *p< 0.05, **p< 0.01, ***p< 0.001.

**Fig. 3:**
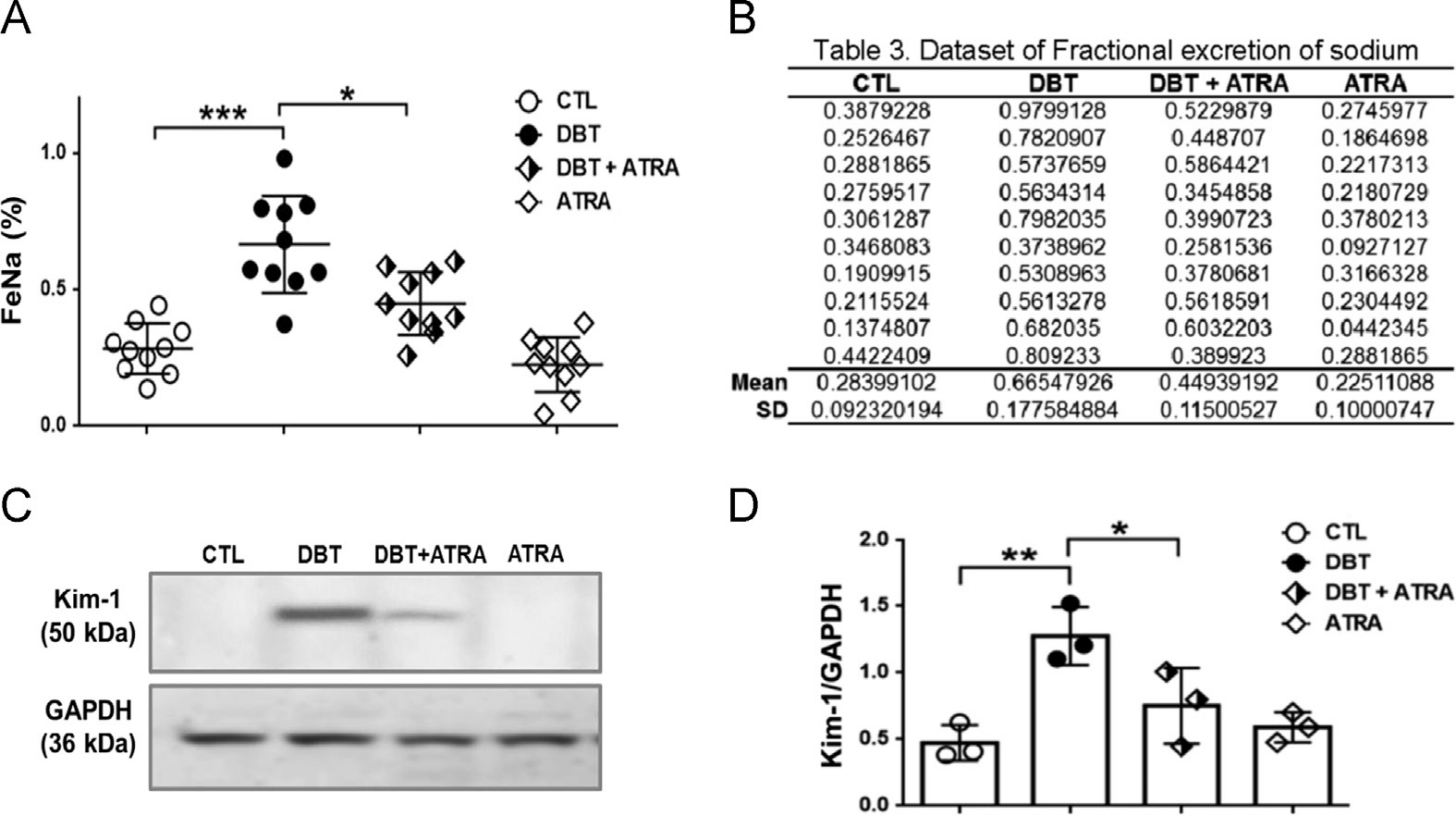
The effects of ATRA in tubule injury in an initial stage of DN were determined using (A) Fractional excretion of sodium (FeNa) *n* = 10, (B)) Raw data used for graph of FeNa, (C) Protein expression by Western blot and (D) densitometric analyses of KIM-1 (early biomarker for DN) in isolated proximal tubules. Glyceraldehyde 3 phosphate (GAPDH) was used as loading control. Data are mean ± SD of 3 independent experiments *p< 0.05, **p< 0.01, ***p< 0.001.

**Fig. 4:**
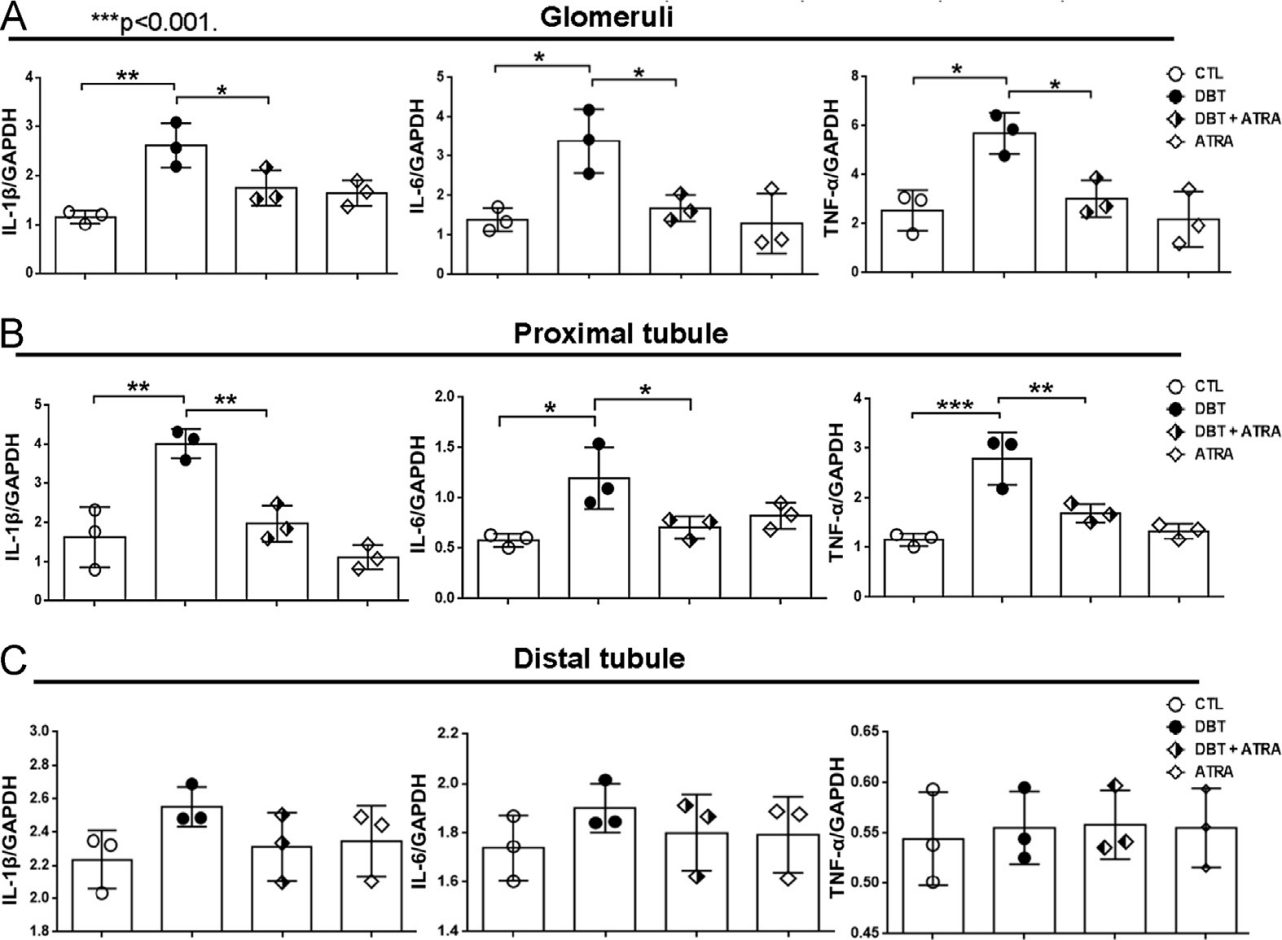
The effect of ATRA in early inflammation in (A) Glomeruli, (B) Proximal and (C) Distal tubules in STZ -induced diabetes. Western blot analyses of IL-1β, IL-6 and TNF-α are shown. GAPDH was used as loading control. Results are expressed as mean±SD of 3 independent experiments *p< 0.05, **p< 0.01, ***p< 0.001.

Inflammation play central role in the progression of DN that lead to renal failure. Our previous study demonstrated that early diabetic condition has a relationship with inflammatory response mediated by TLR4/NF-кB signaling in glomeruli and proximal tubules, respectively, *in vivo* [1]; and, these findings are consistent with previous study which reported that Inflammatory molecules in DN-induced renal injury [2]. The retinoid system plays a key role in maintaining the normal renal structure and attenuates the development of renal pathological changes [3]. The dataset presented in this paper provide information about nephroprotective role of ATRA (Fig. 1, Fig. 2, Fig. 3) and its effect on inflammatory molecules induced by diabetes in the kidney (Fig. 4). We analyzed the effects of its administration in isolated nephron segments: glomeruli, proximal and distal tubules to define their intrarenal selectivity in an early stage of experimental DN.

## 2 Experimental design, materials and methods

### 2.1 Experimental design

Wistar rats were obtained from the production and experimentation unit of laboratory animals (UPEAL) of CINVESTAV-IPN. All animal experiments were performed in accordance with the Mexican Official Norm NOM-062-ZOO-1999, and approved by the UPEAL guidelines (protocol # 0178-16). The rats were divided into four groups of 10–12 animals in each group: a) Control group (CTL), normal rats received a single injection of citrate buffer; pH 4.5 (vehicle); b) Diabetic group (DBT), treated with a single tail-vein injection of streptozotocin (STZ) 60 mg/kg bw, in citrate buffer; pH 4.5); c) Diabetic group treated with ATRA (DBT+ATRA), rats received ATRA (1 mg/kg) once a day intragastrically, until the end of the study (from days 3 to 21 after STZ injection), and d) ATRA group (ATRA), normal control rats received ATRA (1 mg/kg) given orally once a day from days 3 to 21 after single injection of citrate buffer. Rats were sacrificed 21 days after STZ or vehicle administration.

### 2.2 Biochemical and physical studies

Blood glucose and body weight were monitored at days 3, 7, 14 and 21 of the study. The blood glucose was determined by using a glucometer (One Touch® Ultra blood glucose meter). At day 21, blood was collected by cardiac puncture under anesthesia with sodium pentobarbital (30 mg/kg, i.p.) and serum was separated.

### 2.3 Renal function markers

At the end of the experimental period of 3 weeks, rats were housed in metabolic cages for 24 h to collect urine samples. Glomerular filtration rate (GFR) was evaluated through the creatinine clearance. It was calculated with the standard formula [4], and urinary and serum creatinine, were measured by modified Jaffé method. Proteinuria/creatininuria ratio was obtained by dividing urine protein concentration by urine creatinine concentration. Total urinary protein was determined by Lowry method (Bio-Rad Protein Assay Kit, CA, USA). Proximal tubular function was assessed through the urinary and serum sodium concentrations (measured by atomic absorption spectrophotometry) and fractional excretion of sodium (FeNa) was calculated with the following equations: FeNa% = Sodium clearance/creatinine clearance x100.

### 2.4 Isolation of glomeruli, proximal and distal tubules

Glomeruli were isolated by gradual sieving techniques and suspensions of enriched populations of proximal and distal tubules were isolated from renal cortex slices by Percoll density-gradient centrifugation, as previously described [5].

### 2.5 Western blot analyses

Protein samples were extracted from enriched suspensions of nephron sections and used for Western blot studies. Equivalent amounts of proteins were fractionated by sodium dodecyl sulfate polyacrylamide gel electrophoresis (SDS-PAGE) and then transferred onto polyvinylidene difluoride (PVDF) membranes (Millipore, Bedford, MA, USA). After blocking with 5% casein in PBS-buffered saline solution with 0.05% Tween-20, the membranes were immunoblotted overnight al 4 °C with polyclonal antibodies against Kidney Injury Molecule-1 (Kim-1, 1:1000); Interleukin 1 (IL-1, 1:500); Interleukin 6 (IL-6, 1:500) and Tumor necrosis factor alpha (TNF-α, 1:1000). The membranes were incubated with appropriate secondary antibodies and the protein blots on the membranes were visualized by an enhanced chemiluminescence system (UVP BioImaging Systems, Cambridge, UK).
