# Supplementary figures and images for: Data on nephroprotective effect of all-trans retinoic acid in early diabetic nephropathy
Source: Data Brief. 2018 Aug 29;20:784–9. doi: 10.1016/j.dib.2018.08.080 (PMC6129741; doi:10.1016/j.dib.2018.08.080)

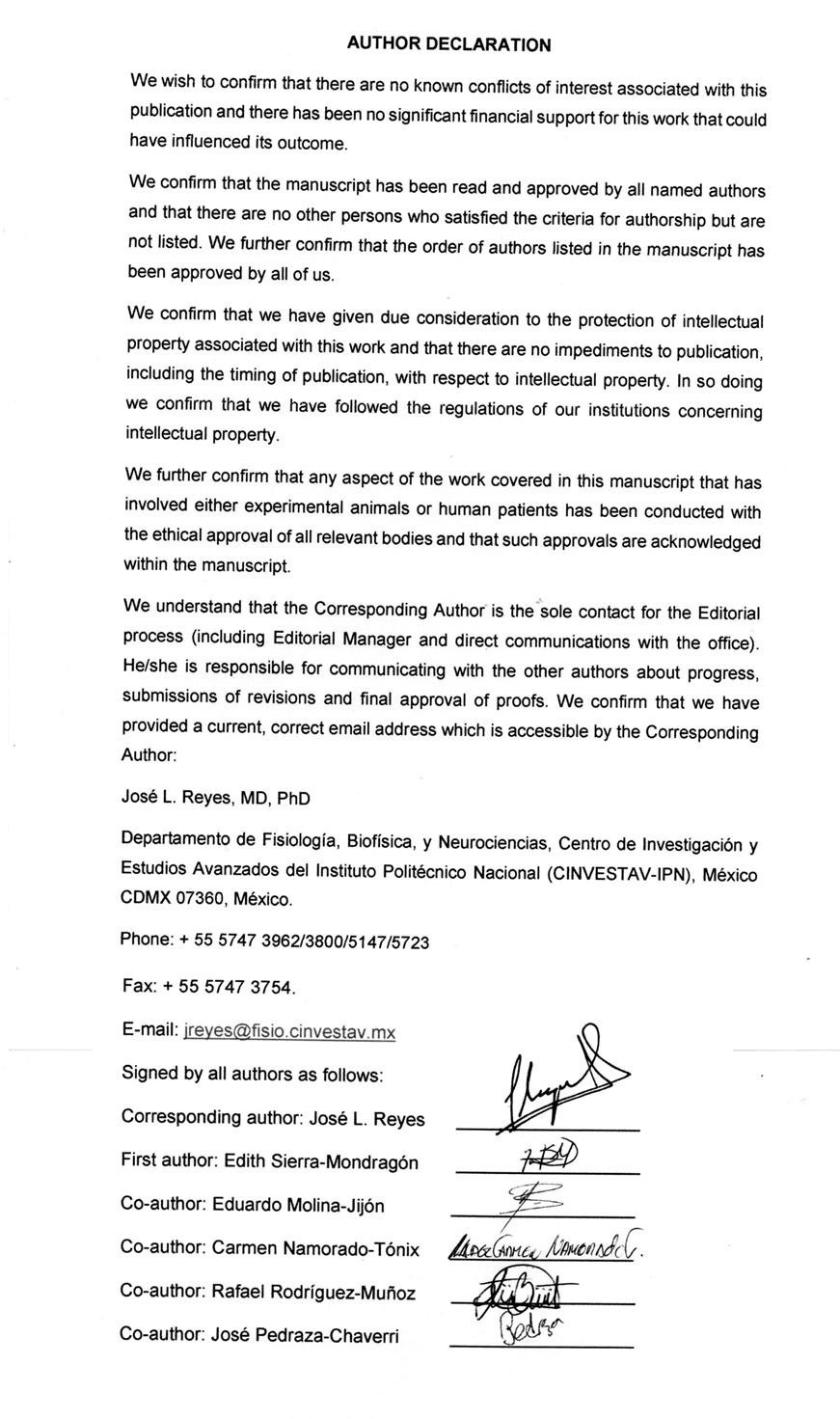

Supplement: Supplementary file 1 — Supplementary material [file mmc1.docx]
